# Annual Migration of Cabbage Moth, *Mamestra brassicae* L. (Lepidoptera: Noctuidae), over the Sea in Northern China

**DOI:** 10.1371/journal.pone.0132904

**Published:** 2015-07-15

**Authors:** Xiao Wu, Xiaowei Fu, Jianglong Guo, Xincheng Zhao, Kongming Wu

**Affiliations:** 1 State Key Laboratory for Biology of Plant Diseases and Insect Pests, Institute of Plant Protection, Chinese Academy of Agricultural Sciences, Beijing, 100193, PR China; 2 Department of Entomology, College of Plant Protection, Henan Agricultural University, Zhengzhou, 450002, China; CNRS, FRANCE

## Abstract

The cabbage moth, *Mamestra brassicae* L. (Lepidoptera: Noctuidae), is a serious pest of vegetable crops throughout the world. In order to determine whether or not *M*. *brassicae* is a migrant, and if yes, what is the pattern of *M*. *brassicae* seasonal migration, a long-term study on *M*. *brassicae* from April to October in 2003–2014 was carried out by means of a searchlight trap on a small island located in the center of the Bohai Strait. The results show that a large number of *M*. *brassicae* were trapped every year on the island, which indicates that *M*. *brassicae* is a migrant and migrated at least 40–60 km across the Bohai Strait. The mean migration period of *M*. *brassicae* over the sea within one year is 151 ± 8 d in 2003–2014, with the shortest time span 78 d in 2003 and the longest 189 d in 2014, respectively. The number of *M*. *brassicae* captured, however, varies considerably between months or years. The majority of captures were female, with different levels of ovarian development and mating status. Most of the females trapped in May-July during 2010–2014 had a high mating rate and advanced level of ovarian development, suggesting that the migration of this species does not conform to the hypothesis of ‘oogenesis-flight syndrome’. The findings of the present study are beneficial to the development of forecasting systems and management strategies of *M*. *brassicae*.

## Introduction

The cabbage moth, *Mamestra brassicae* L. (Lepidoptera: Noctuidae), is a serious pest species of vegetable crops with a widespread distribution around the world, mainly including the regions from 30°N to about 70°N of Europe and Asia [1–4]. The *M*. *brassicae* has 1–4 generations each year from the northern area to the southern [2,5,6], and its occurrence exhibits intermittent and outbreak characteristics [7]. The *M*. *brassicae* is a highly polyphagous insect with a wide host range (more than 300 species of 100 families), of which the Brassicaceae is the most preferred [4,8]. In general, this pest causes about 20%-25% of the damage rate of vegetables and 25%-40% reductions in yield each year [9].

Migration is the seasonally synchronized movement of insects, with a regular pattern or a route to link temporary breeding sites at a much greater scale and longer duration than its normal daily activities, which leads to redistribution with a spatially extended population [10]. Lots of insect species migrate poleward from lower-latitude winter habitats each spring to places where they can breed during the summer but can’t overwinter successfully [11,12]. Insects, with the most abundant biodiversity, play an important role in many aspects, e.g. pollination, biological control, physical decomposition and providing a wide range of products [13]. Billions of insects migrate yearly, which provides major ecosystem services, causing serious crop damage, and spreading diseases of humans and their livestock [12].

In the present study, long-term (12-year) observations of the seasonal migration of *M*. *brassicae* across the Bohai Sea were carried out with a searchlight trap on a small island located in the center of the Bohai Strait. The objectives of the long-term observations are to determine (1) whether or not the *M*. *brassicae* is a migrant; (2) if yes, what is the pattern of *M*. *brassicae* seasonal migration? (3) and then whether the migrations are regular occurrences or incidental events. Understanding the migration of *M*. *brassicae* will increase our knowledge of the outbreaks of *M*. *brassicae* in East Asia, and facilitate the development of forecasting systems and management strategies for *M*. *brassicae*.

## Materials and Methods

### Searchlight trapping and field observation

**Fig 1:**
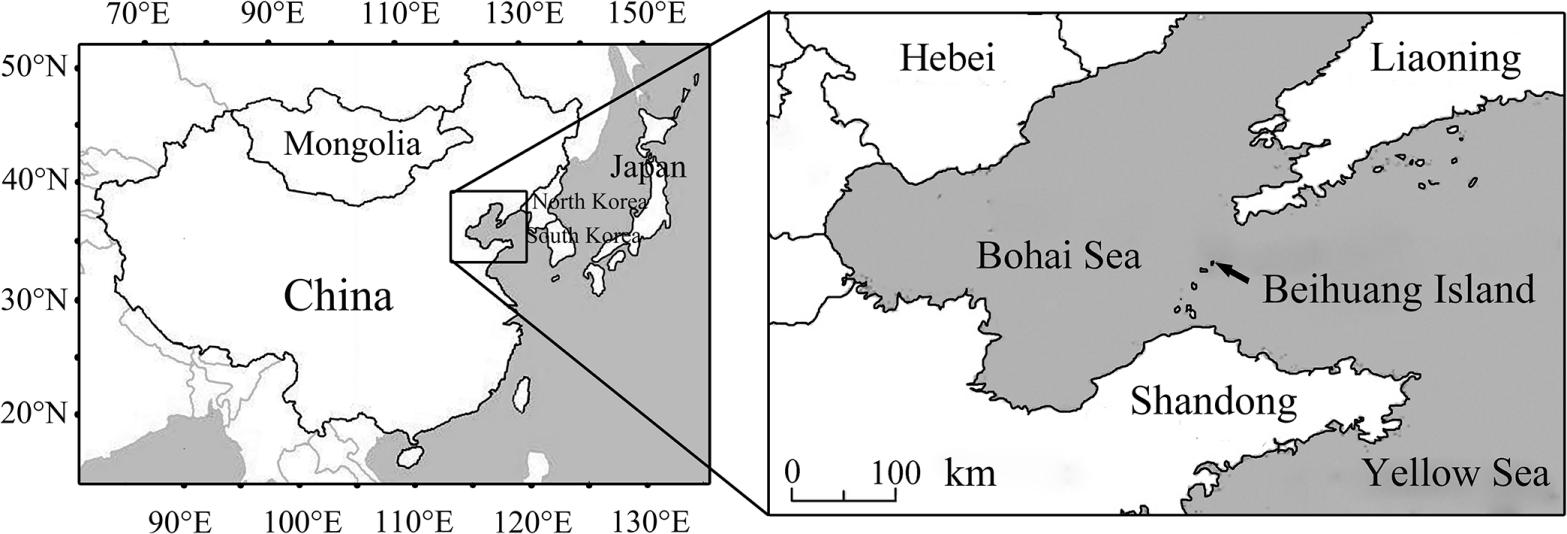
Maps showing the area of East Asia (left-hand map) and the position of Beihuang Island, the searchlight trap site (right-hand map).

The searchlight trapping studies were carried out from April to October in 2003–2014, at Beihuang (BH) island (38°23.200´N; 120°54.500´E), which was located in the center of the Bohai Gulf at a distance from the mainland of about 60 km to the south and about 40 km to the north (Fig 1). No specific permissions were required for the study on this island, because the Chinese Academy of Agricultural Sciences is an affiliated part of the Ministry of Agriculture of the People’s Republic of China. We confirm that the field studies did not involve endangered or protected species. For attracting and capturing high-altitude migrants (up to 500 m above ground level) [14], a vertical-pointing searchlight was used, which was equipped with a 1,000 W metal halide lamp (model JLZ1000BT; Shanghai Yaming Lighting Co. Ltd., Shanghai, China), producing a vertical beam of light with a luminous flux of 105,000 lm, a color temperature of 4,000 K, and a color rendering index of 65 [15].

The searchlight trap was turned on at sunset (from 17:15 in 31^st^ October to 19:30 in 22^nd^ June, UTC +8) and turned off at sunrise (from 4:45 in 22^nd^ June to 6:40 in 31^st^ October, UTC +8) on every night from April to October 2003–2014, except when it was raining or when power was cut (incomplete data sets caused by power cuts or heavy rains were excluded from the following analysis; while, those nights in which the light trapping was carried out normally but no *M*. *brassicae* was captured, ‘zero’ was used in the following analysis). Trapped insects were collected with a nylon net bag (60 mesh) beneath the trap, which was renewed manually every 2 h each night. The trapped insects were kept in a freezer at -20°C for 4 h before being identified. The reported results in this paper were from a single trap, and that the additional two traps during the 2013–2014 seasons did not yield different results both in the trapped number and species composition, so they were not presented.

There are no arable lands and host crops of *M*. *brassicae* on BH. To verify whether *M*. *brassicae* originally existed on BH, visual observations were carried out daily on any potential wild host plants to detect *M*. *brassicae* larvae from spring to autumn during 2003–2014.

### Ovarian dissection

A subsample of 20 females (or all individuals if the total capture of females was less than or equal to 20) was randomly taken from adults trapped each night from 2010 to 2014, and dissected with adding of a stereomicroscope (model JNOEC-Jsz4; Motic China Group Co. Ltd., Xiamen, China). The level of ovarian development 1–5 was estimated according to the criteria described in Table 1. These data were used to generate an average monthly level of ovarian development (i.e., the sum of individual levels of ovarian development divided by the number of females dissected). Females with ovarian development level 1–2 were regarded as “sexually immature individuals,” and others with level 3–5 were regarded as “sexually matured individuals” [16,17]. Moreover, mating frequency and mating occurrences of *M*. *brassicae* were determined by the number of spermatophores in the female spermatheca. Mating rate is the proportion of mated females (i.e., the number of mated females divided by the number of females examined).

**Table 1:** Criteria of ovarian development level of *M*. *brassicae* adults (references [15,16]).

Development level	Ovarian characteristics
**1**	Undeveloped thin and short oviducts, with transparent and light milky white ovary
**2**	Developed longer oviducts, with developing oocytes but no mature eggs
**3**	Well-developed yellowish green ovary with 1–3 chorionated eggs stored in the egg calyx
**4**	Well-developed the biggest oviducts, some eggs have been laid and interspaces appeared among matured eggs
**5**	The ovary has atrophied and contains no eggs

### Data analysis

Dates of trapping catches reported in this paper indicate the period from sunset of that day to sunrise of the next day, and means are given as means ± SEM. The nightly mean catches of *M*. *brassicae*, the mean proportion of females, the average mating rate, and the mean proportion of sexually mature females were analyzed by one-way analysis of variance (ANOVA), with month as the fixed effect and year as the random effect (PROC ANOVA). If the ANOVA indicated a significant difference, Tukey's HSD (honestly significant difference) test was followed to separate the means. Differences in the mean proportion of mated and unmated females, and in the mean proportion of sexually mature and immature females in each month from 2010 to 2014 were analyzed by t-tests (PROC TTEST), and the temporal autocorrelation of the sum of captured *M*. *brassicae* during 2003–2014 was analyzed by autocorrelation tests. These three kinds of statistical analyzes were performed with SPSS software [18]. All the proportion data were mean square root arcsine transformed before processed. The sex ratio (females: males) in each month from 2010 to 2014 was compared to a ratio of 1:1 using chi-square tests (PROCFREQ) with SAS software [19]. Raw data and the results of analysis were also presented in S1 Dataset as supporting information.

## Results

### Annual and monthly pattern of migration

**Fig 2:**
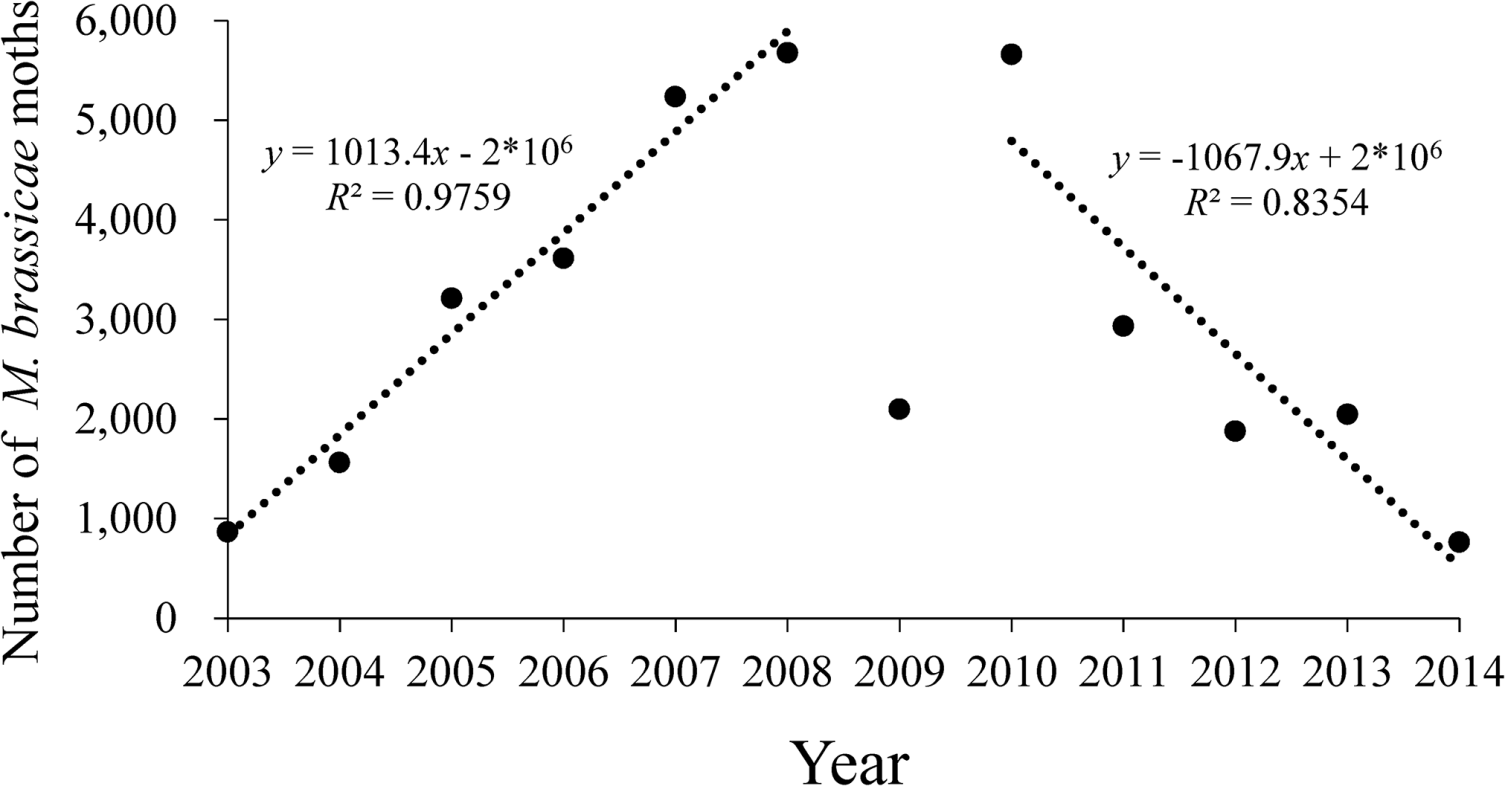
Annual catches of *M*. *brassicae* in the searchlight trap on BH from 2003 to 2014. Linear model (dotted lines): *y* = 1013.4*x*-2×10^6^; *R* ^2^ = 0.9759 (2003–2008); *y* = -1067.9*x*+2×10^6^; *R* ^2^ = 0.8345 (2010–2014).

**Fig 3:**
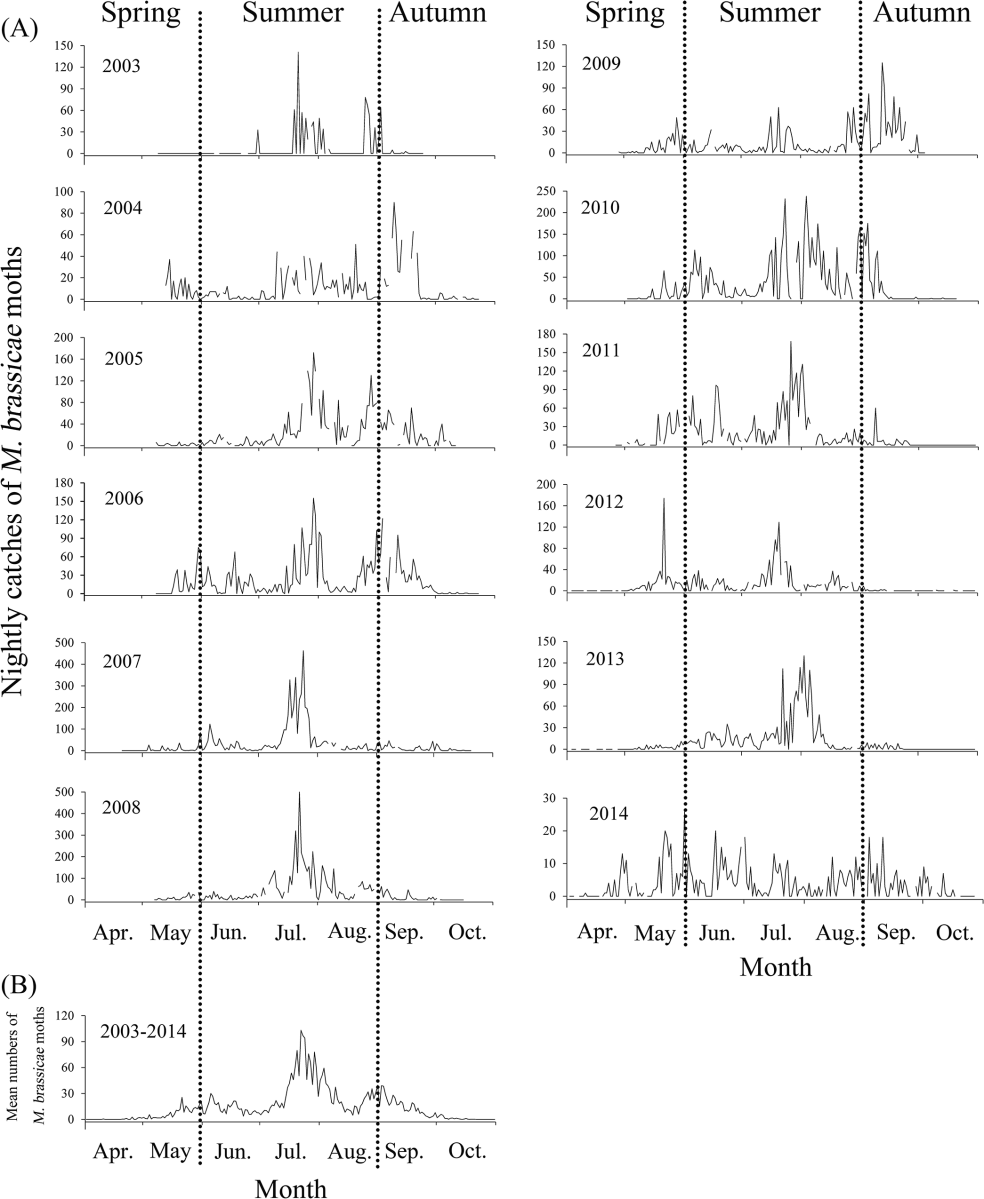
Nightly catches (A) and mean numbers in the twelve years (B) of *M*. *brassicae* in the searchlight trap on BH from April to October during 2003–2014. The dashed vertical lines represent the separation between seasons.

Although some wild cruciferous weeds were available as potential hosts, field investigation found no *M*. *brassicae* larvae on BH. The catches in BH searchlight traps showed considerable abundance of *M*. *brassicae* from spring to autumn during 2003–2014 (Figs 2 and 3). The number of captured *M*. *brassicae* differed between years. The total number of annual catches of *M*. *brassicae* increased significantly from 899 to 5676 individuals during 2003–2008, which showed a steadily rising trend (linear model, *y* = 1013.4*x*-2×10^6^; *R* ^*2*^ = 0.9759; *F* = 162.137; *P* < 0.0001; Fig 2). However, the number of *M*. *brassicae* suddenly reduced to 2097 individuals in 2009. Then the yearly amount of trapped *M*. *brassicae* reduced significantly to show a downward trend from 2010 to 2014 (linear model, *y* = -1067.9*x* + 2×10^6^; *R* ^*2*^ = 0.8354; *F* = 15.226; *P* = 0.030; Fig 2). There is no temporal autocorrelation about the yearly totals of trapped *M*. *brassicae* during 2003–2014 (S1 Dataset).

In this decade, *M*. *brassicae* were first captured in the searchlight trap generally in late-April and early-May, and the earliest individual was captured on 10 April 2014 (Table 2; Fig 3A and 3B). Thereafter, the moths were trapped intermittently rather than consecutively on every night, until late May during the twelve years. After mid-July, mean catches of *M*. *brassicae* per night increased and reached upsurge maximum in late-July. The upsurge formed an obvious migration wave in late-July, which then decreased to a low level with a migration trough in mid-August. However, searchlight trap captures increased from late-August to early-September, showing another small-scale migration wave at the intersection of the two months (Fig 3A and 3B). From mid-September, *M*. *brassicae* were trapped intermittently, which led to a low level of nightly mean catches in late-September and no catches in mid-October (Fig 3A and 3B).

**Table 2:** Dates of first and final capture of *M*. *brassicae* and duration of capture period in the twelve years in the searchlight trapping on BH.

Year	Date of first capture (n)^a^	Date of final capture (n)^a^	Duration of capture (d)	Date of peak catches (n)^a^
**2003**	30-June (33)	16-September (1)	78	21-July (141)
**2004**	13-May (13)	20-October (1)	160	09-September (90)
**2005**	08-May (6)	08-October (8)	153	29-July (172)
**2006**	17-May (12)	17-October (1)	153	29-July (155)
**2007**	04-May (26)	16-October (2)	165	24-July (463)
**2008**	07-May (1)	04-October (1)	150	22-July (500)
**2009**	28-April (1)	30-September (25)	155	12-September (125)
**2010**	08-May (2)	13-October (2)	158	03-August (238)
**2011**	02-May (4)	25-September (4)	146	26-July (168)
**2012**	03-May (1)	19-October (1)	169	21-May (174)
**2013**	06-May (1)	22-September (1)	139	02-August (130)
**2014**	10-Apr (1)	16-October (1)	189	31-May (26)

^a^ The numbers of *M*. *brassicae* moths captured are given in parentheses next to the date of capture.

There was considerable variation (*F* = 7.088, *df*_1_ = 6, *df*_2_ = 70, *P* < 0.001) in the number of *M*. *brassicae* captured between months during 2003–2014, with 7.63 ± 3.7% trapped in spring (April-May), 77.41 ± 7.91% trapped in summer (June-August), and 14.96 ± 6.75% trapped in autumn (September-October) (Fig 3A and 3B).

The mean period of catching *M*. *brassicae* on BH within one year was 151 ± 8d from 2003 to 2014, with the shortest time span 78 d in 2003 and the longest 189 d in 2014, respectively (Table 2).

### Sex ratio, mating frequency, and ovarian development

**Fig 4:**
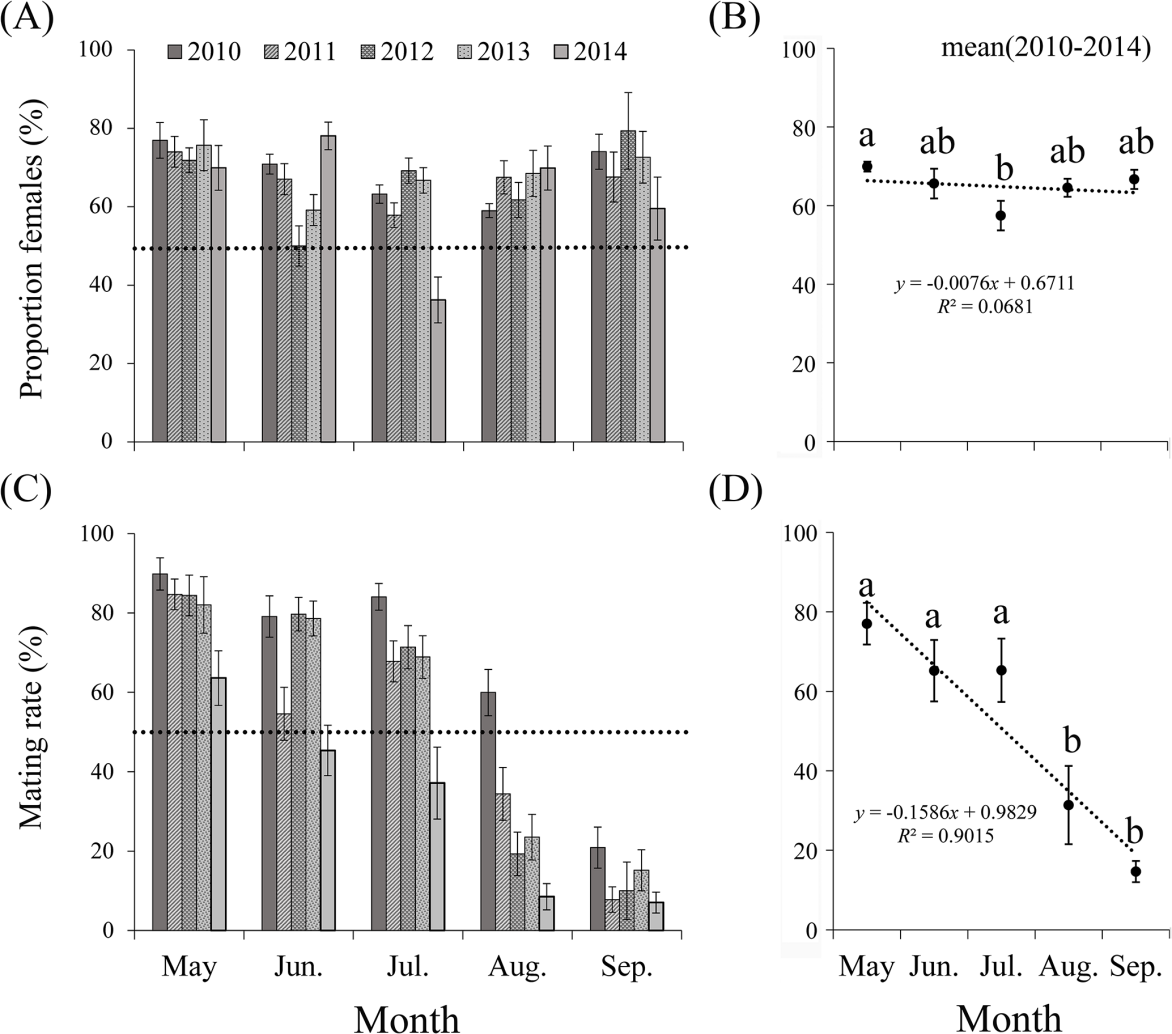
Proportion of females (A-B) and mating rate (C-D) of *M*. *brassicae* captured in the searchlight trap on BH from May to September during 2010–2014. The histograms in A and C indicate average daily proportion in each month, and the vertical bars represent standard errors between days in that month. The scatter diagrams in B and D indicate average proportion from 2010 to 2014 in each month, and the vertical bar represent standard errors between years in that month. Bars sharing the same letter mean there were no significant differences during months at the 5% level by Tukey’s HSD tests. Linear model (dotted lines): (B) *y* = -0.0076*x*+0.6711; *R* ^2^ = 0.0681; (D) *y* = -0.1586*x*+0.9829; *R* ^2^ = 0.9015. Few moths were trapped in April and October, so these months are not presented.

From May to September during 2010–2014, the majority of trapped *M*. *brassicae* was female (Fig 4A and 4B). Chi-square tests showed that the proportion of trapped females was significantly higher than that of males in all months (May, *χ* ^*2*^ = 191.49, *df* = 1, *P* < 0.0001; June, *χ* ^*2*^ = 245.39, *df* = 1, *P* < 0.0001; July, *χ* ^*2*^ = 192.71, *df* = 1, *P* < 0.0001; August, *χ* ^*2*^ = 190.34, *df* = 1, *P* < 0.0001; September, *χ* ^*2*^ = 96.16, *df* = 1, *P* < 0.0001; *χ* ^*2*^ = 191.49, *df* = 1, *P* < 0.0001). There was no significant difference (*F* = 2.54, *df*_*1*_ = 4, *df*_*2*_ = 20, *P* = 0.072) in the mean proportion of females between months, which show a weak downward trend (linear model, *y* = -0.0076*x*+0.6711; *R* ^*2*^ = 0.0681; *F* = 0.219; *P* = 0.671) from May to September (Fig 4B).

**Fig 5:**
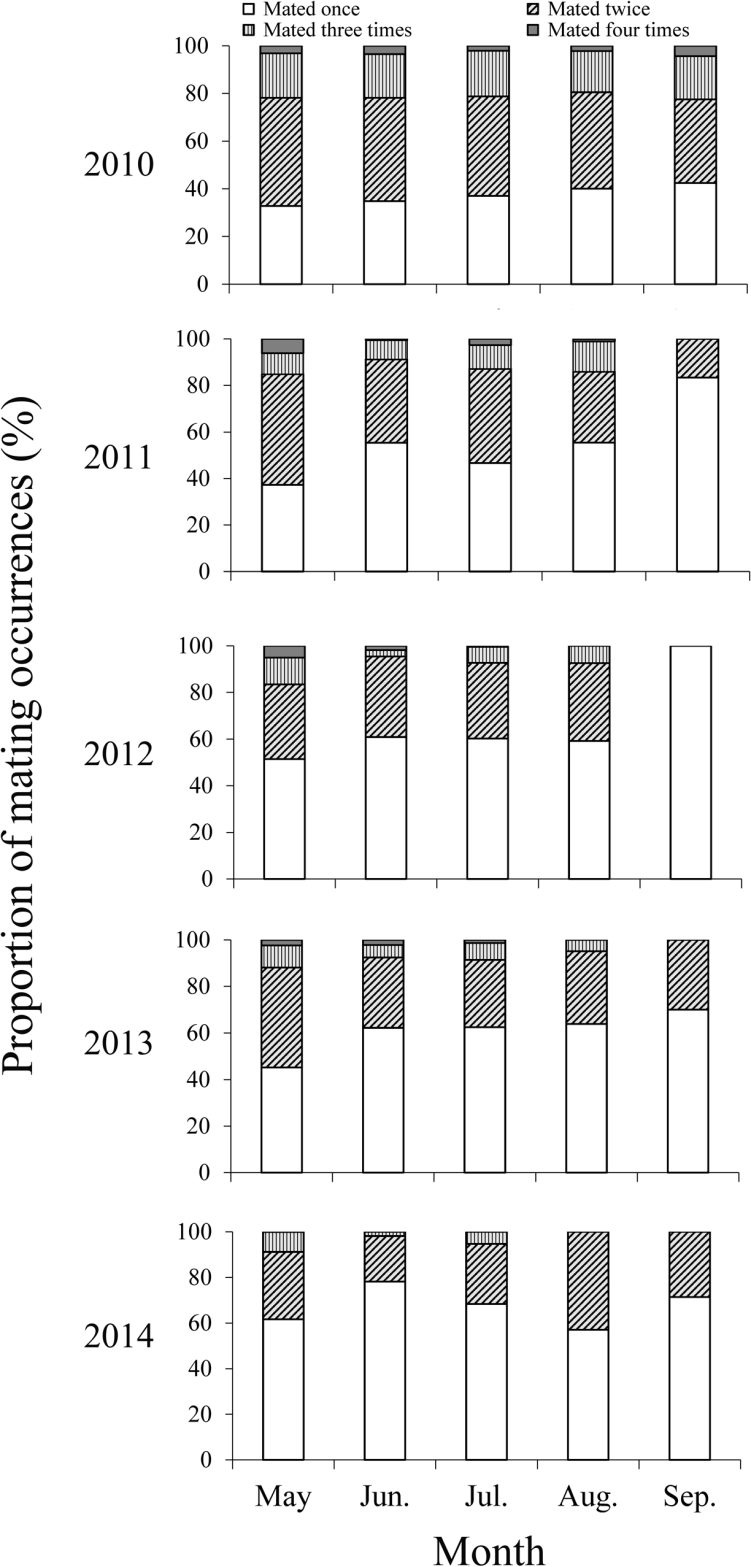
Proportion of mating occurrences of *M*. *brassicae* females captured in the searchlight trap on BH from May to September during 2010–1014. Few moths were trapped in April and October, so these months are not presented.

Most trapped female migrants were mated individuals in late-spring (May), the mean proportion of mated females reached 77.03 ± 5.24%, and t-tests showed that it was significantly (May: *t* = 4.99, *df* = 4, *P* = 0.008) higher than that of unmated individuals (Fig 4C and 4D). In summer (June, July and August), the mean mating rate reached 65.20 ± 7.74%, 65.30 ± 7.97% and 31.38 ± 9.85%, respectively. There was no significant difference (June: *t* = 1.97, *df* = 4, *P* = 0.12; July: *t* = 1.93, *df* = 4, *P* = 0.125; August: *t* = -1.896, *df* = 4, *P* = 0.131) between the proportion of mated and unmated females (Fig 4C and 4D). However, the vast majority of trapped females in September were virgins, and t-tests showed that the proportion of mated females was significantly (September: *t* = -10.40, *df* = 4, *P* < 0.0001) lower than that of unmated females (Fig 4C and 4D). Overall, there was significant difference (*F* = 13.849, *df*_*1*_ = 4, *df*_*2*_ = 20, *P* < 0.0001) in the average ratio of mated females between months, showing a significant downward trend (linear model, *y* = -0.1586*x*+0.9829; *R* ^*2*^ = 0.9015; *F* = 27.456; *P* = 0.014) from May to September (Fig 4D). During 2010–2014, most mated females had mated once or twice, and only a few had mated three or four times from May to September (Fig 5).

**Fig 6:**
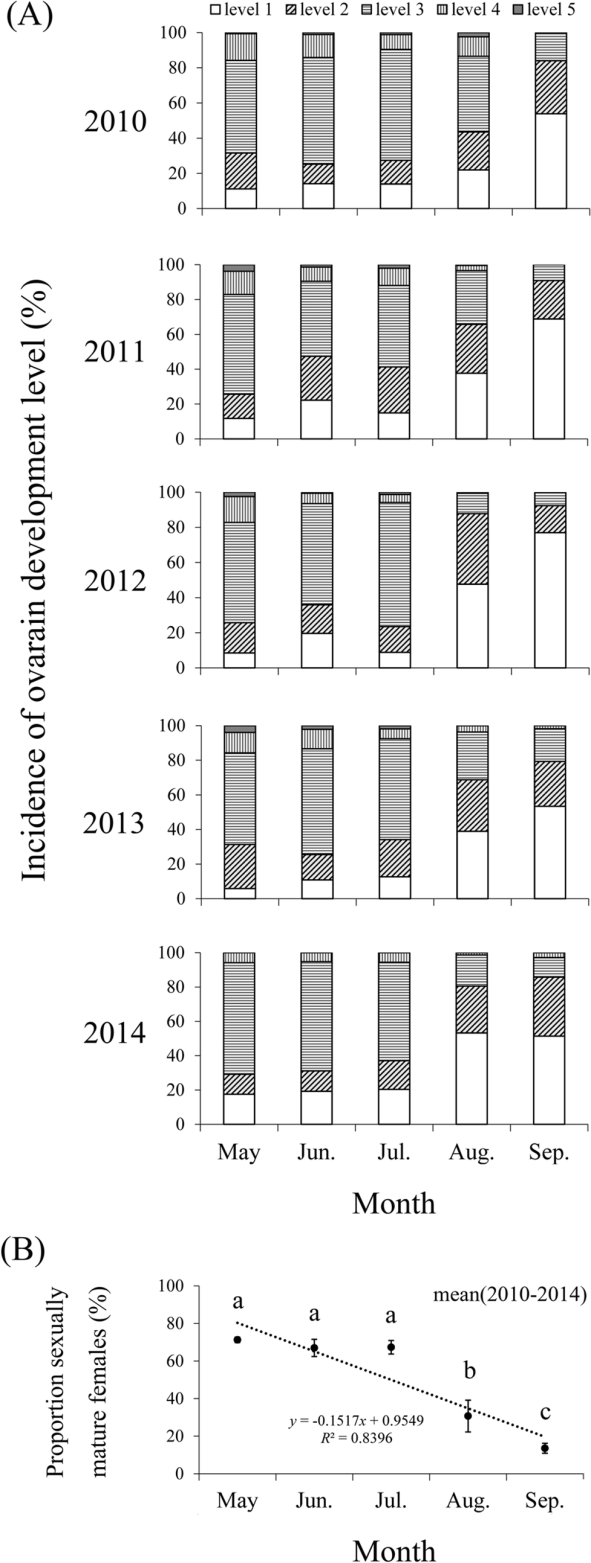
Incidence of ovarian development (A) and mean proportion of sexually mature females (B) of *M*. *brassicae* captured in the searchlight trap on BH from May to September during 2010–2014. The scatter diagrams in B indicate average proportion of sexually mature females from 2010 to 2014 in each month. Bars sharing the same letter mean there were no significant differences during months at the 5%level by Tukey’s HSD tests. Linear model (dotted lines): (B) *y* = -15.165*x*+95.459; *R* ^2^ = 0.8394. Few moths were trapped in April and October, so these months are not presented.

From 2010 to 2014, only a few *M*. *brassicae* females with ovarian development level 5 were found on BH (Fig 6A). Most of the captured females from May to July showed a certain degree of ovarian development, and the proportion of sexually mature females reached 71.26 ± 1.45%, 66.96 ± 4.59%, and 67.33 ± 3.59%, respectively. The mean proportion of sexually mature females in the summer was significantly (May: *t* = 15.34, *df* = 4, *P* < 0.0001; June: *t* = 4.06, *df* = 4, *P* = 0.015; July: *t* = 5.16, *df* = 4, *P* = 0.007) higher than that of sexually immature females. In August, there was no significant difference (August: *t* = -2.50, *df* = 4, *P* = 0.067) between the mean proportion of sexually mature and immature females. However, the proportion of sexually mature females decreased to low levels, which was significantly (September: *t* = -11.83, *df* = 4, *P* < 0.0001) lower than that of sexually immature females in September (Fig 6B). At the same time, there was significant inter-month difference (*F* = 35.44, *df*_*1*_ = 4, *df*_*2*_ = 20, *P* < 0.0001) in the mean proportion of sexually mature females from 2010 to 2014, showing a significant downward trend (linear model, *y* = -15.165*x*+95.459; *R* ^*2*^ = 0.8394; *F* = 15.706; *P* = 0.029) from May to September (Fig 6B).

## Discussion

During 2003–2014, the results of searchlight trapping showed that there was a large number of captured *M*. *brassicae* on BH every year, which provided the first conclusive evidence that *M*. *brassicae* is a migrant species. Meanwhile, the *M*. *brassicae* might have migrated at least 40–60 km across the Bohai Strait to reach the trapping site. The frequently captured *M*. *brassicae* during the twelve years, suggests that over-sea migration of this species has become the regular ecological behavior. The population dynamics of *M*. *brassicae* in this study were similar to previous observations in other species of Lepidoptera, Odonata, Hemiptera and Coleoptera made on this island [14,20–26]. Our results showed that there was a continuous capture of *M*. *brassicae* during 2003–2014, and huge number of catches occurred separately in 2007, 2008 and 2010. Although there was no regular monitoring of this pest in the mainland, we could not exclude the possibility that the quantity change may reflect the occurrence of this pest in the mainland. If so, our monitoring on the island provided good surveillance of this pest species.

From May to September, the proportion of trapped *M*. *brassicae* females per month was significantly higher than that of males. In late-spring (May) and early-summer (June and July), the mean mating rate and the index of ovarian development of *M*. *brassicae* females were significantly higher than in other months, which might be due largely to long-distance migration *M*. *brassicae* to the island during several continuous nights. Active flight leads to a significant body temperature rise and juvenile hormone biosynthesis [27–29]. Combining with these aspects, it is foreseen that a certain degree of sexual maturation or mating would occur within several days of initiating migration, and the onset of maturation would be conducive to the female migrants, after finding proper food resources and habitat, they could mate and oviposit as early as possible [30]. Meanwhile, the high mating rate and advanced level of ovarian development of *M*. *brassicae* during this period indicate that the migration of this species is not suppressed by the onset of adult ovarian development and mating status, which does not conform to the hypothesis of oogenesis-flight syndrome [31–33]. It is possible that the development of the flight system and ovary of *M*. *brassicae* is synchronous, and this condition is similar to *Mythimna separata* W. and *Euxoa auxiliaris* [34,35].

However, in August and September, female migrants of *M*. *brassicae* have a relatively lower mating rate and ovarian development, proving that the onset of migration begins with sexually immature individuals [36]. Sexually immature individuals trapped during this period may be due to short-distance migration of *M*. *brassicae* to the trapping site. This case is similar to that of many other migratory insects, such as *Agrotis ipsilon* R. in North America and *Plutella xylostella* L. in China [24,37].

The East Asian monsoon airflows in the temperate regions play a major role in long-distance insect migration [38]. In summer, there are prevailing southwesterly winds in the coastal regions of southern China and southeasterly winds in the regions of East Asian continent-Japan-Korea, while in winter, there are prevailing northeasterly winds in the south of 30°N and northwesterly winds in the north of 30°N [39,40]. The combination of radar and meteorological studies demonstrated that migrations of *H*. *armigera* and *Loxostege sticticalis* L. are coincident with changes of the East Asian monsoon [41–43].

In the current study, the population dynamics of *M*. *brassicae* were roughly coincident with changes of the East Asian monsoon. It is possible that migrating *M*. *brassicae* are transported for long distances by wind. In late-spring (May) and early-summer (June and July), *M*. *brassicae* may migrate into north-eastern China or even further north (namely Mongolia) by summer monsoon to exploit widespread habitats. In late-summer and early-autumn, this species may move back into central and southern China by winter monsoon to overwinter there or breed a generation. In order to verify such hypothesis, however, further studies combining population dynamics with radar detection, meteorological elements, and migrant trajectory analysis, are necessary to be performed on *M*. *brassicae*.

## Supporting Information

S1 Dataset All data including the raw data and the results of analysis that underlying the findings of this study were presented in S1 Dataset as supporting information. (XLSX)
